# The Influence of the Presence of the Ramus Intermedius on Atherosclerosis Plaque Deposition in the Left Bifurcation Region in Low-Risk Individuals

**DOI:** 10.31083/RCM25252

**Published:** 2025-02-13

**Authors:** Nurul Sazmi Rosani, Rasheeda Mohd Zamin, Raja Rizal Azman Raja Aman, Ahmad Syadi Mahmood Zuhdi, Mahmoud Danaee, Intan Suhana Zulkafli

**Affiliations:** ^1^Department of Anatomy, Faculty of Medicine, Universiti Malaya, 50603 Kuala Lumpur, Malaysia; ^2^Department of Anatomy, Faculty of Medicine, Universiti Teknologi MARA, Sungai Buloh Campus, 47000 Selangor, Malaysia; ^3^Department of Biomedical Imaging, University Malaya Medical Centre, 59100 Kuala Lumpur, Malaysia; ^4^Department of Medicine, University Malaya Medical Centre, 59100 Kuala Lumpur, Malaysia; ^5^Department of Social and Preventive Medicine, Faculty of Medicine, Universiti Malaya, 50603 Kuala Lumpur, Malaysia

**Keywords:** atherosclerosis plaque deposition, cardiac computed tomography angiography, left coronary artery, ramus intermedius

## Abstract

**Background:**

Additional bifurcations at the left main coronary artery (LMCA) could modify the geometry of the left coronary system, disturbing haemodynamic flow patterns and potentially altering endothelial shear stress (ESS). A low ESS has been implicated in atherogenesis. The emergence of the ramus intermedius (RI) from the LMCA creates additional branching, but the specific role of the RI in plaque deposition at the left coronary system remains unclear. This study sought to elucidate the potential effects of the RI on plaque formation at the LMCA and its bifurcation.

**Methods:**

A retrospective cross-sectional single-centre study was conducted using data from 139 female patients who were identified to have low risk of cardiovascular disease. These patients underwent cardiac computed tomography angiography between January 2017 and December 2018. Contrasted multiplanar coronary images taken during the best diastolic phase were analysed for the presence (experimental group) or absence (control group) of the RI. Measurements of plaques were done at the LMCA and at a 10 mm distance from the ostia of daughter arteries. Plaque data at the left bifurcation region were analysed using descriptive statistics, chi-square, and binary logistic regression tests. A *p*-value of <0.05 was considered statistically significant.

**Results:**

Amongst these low-risk patients, 33.8% (n = 47) had an RI. In the presence of RI, there was an eight-fold increased risk of plaque deposition at the LMCA (adjusted odds ratio, aOR = 8.5) and a three-fold increased risk of plaque deposition at the proximal left anterior descending (pLAD), especially on its lateral wall (aOR = 3.5). However, the RI did not influence plaque deposition at the distance of 10 mm from the ostium of the proximal left circumflex artery.

**Conclusions:**

These findings suggest that the RI increases the risk for atherosclerosis plaque deposition by three to eight-fold at the pLAD artery and the LMCA.

## 1. Introduction

Alteration in arterial geometry, such as the presence of a bifurcation, epicardial curvature, and additional branch point significantly influences local haemodynamics [1, 2, 3]. These geometric changes affect endothelial shear stress (ESS), a key factor in endothelial injury and play a critical role in promoting atherogenesis [4, 5, 6, 7, 8]. Previous research has found that the configuration of the left bifurcation region (LBR), particularly its wide angulation, is known to disrupt coronary haemodynamics [4, 9, 10, 11]. The ramus intermedius (RI) is an anatomical variant branch that arises from the left main bifurcation angle (LMBA) between the left anterior descending (LAD) and left circumflex (LCx) arteries [12, 13, 14].

Several case reports from Glancy (2017) [15], Birnbaum and Alam (2022) [16], and Khachatryan *et al*. (2024) [17] showed incidental findings of the RI in patients with a history of myocardial infarction. The specific role of the RI in the onset of myocardial infarction remains poorly understood. However, the change of arterial geometry as the result of an additional branch point created by the RI was thought to have a contributory effect [15, 16, 17]. The current study, therefore, sought to analyse the role of the RI in plaque formation at the LBR by examining its association with plaque deposition in low-risk individuals. This research intended to provide clarity on how this anatomical variant may contribute to the pathogenesis of atherosclerosis within the left coronary circulation.

## 2. Materials and Methods

### 2.1 Data Acquisition and Analysis Tools 

#### 2.1.1 Study Population

**Fig. 1.**
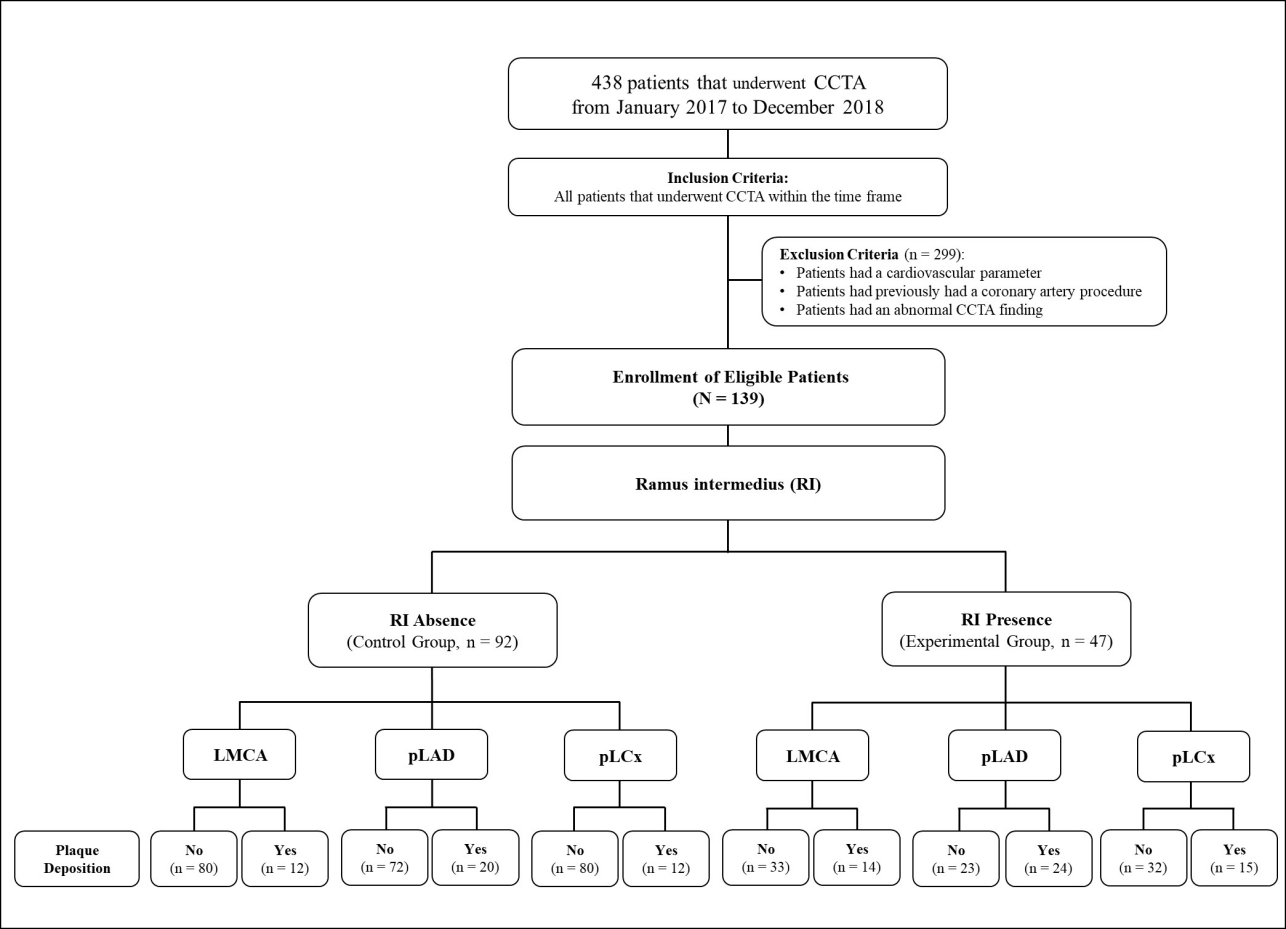
**The study flow chart**. CCTA, cardiac computed tomography angiography; LMCA, left main coronary artery; pLAD, proximal left anterior descending artery; pLCx, proximal left circumflex artery; RI, ramus intermedius.

A single-centre retrospective assessment was conducted at a tertiary teaching hospital in Malaysia, encompassing cardiac computed tomography angiography (CCTA) images of left main coronary arteries (LMCAs) between January 2017 and December 2018. A total of 438 patients who underwent CCTA during this two-year period were included in this study (Fig. 1). After excluding patients according to the exclusion criteria, 139 patients were eligible for study enrolment. Exclusion criteria were: (1) presence of one or more cardiovascular parameters (detailed below), (2) history of coronary interventions (i.e., stenting or bypass surgery), (3) absence of the LMCA, and (4) poor-imaging quality or presence of artefacts in their radiographs.

A total of 139 low-risk individuals were finally included in this study. Low-risk individuals were defined as those who did not fulfil any criteria for positive cardiovascular parameters, such that these patients had no cardiovascular risk factors or cardiovascular diseases. The description of cardiovascular risk factors and diseases was made in accordance with the Management of Chronic Coronary Syndromes (2024) from the European Society of Cardiology and the Guideline on the Primary Prevention of Cardiovascular Disease (2019) by the American College of Cardiology and American Heart Association. These parameters include male gender, a smoking history, diabetes mellitus, hypertension, dyslipidaemia, cerebrovascular disease, and peripheral arterial disease [18, 19].

#### 2.1.2 CCTA Data Acquisition

All CCTA scans were performed using a Somatom Definition Dual Source 64-slice computed tomography scanner (Siemens Healthcare, Forchheim, Germany). The imaging protocol adhered to the Society of Cardiovascular Computed Tomography Guidelines. Patients were fasted overnight and refrained from caffeine and theophylline intake for 24 hours prior to the scan. A beta-blocker was administered if the heart rate exceeded 65 beats per minute [20].

Imaging parameters included slice acquisition of 2 × 32 × 0.6 mm (19.2 mm z-axis coverage), a full tube current between 20% to 75% of R-R interval, a rotation time of 330 ms, a temporal resolution of approximately 82.5 ms with a reference of 320 mAs. A low tube voltage of 120 kV was used for retrospective electrocardiogram-triggered scans with routine contrast protocol dose modulation. Contrast-enhanced CCTA was achieved using 370 mg L/mL (infusion rate of 5 mL/s) of intravenous iopromide. The scans were taken at a 10-second breath-hold, and the images were captured during the best-diastolic phase.

#### 2.1.3 Image Reconstruction and Analysis

**Fig. 2.**
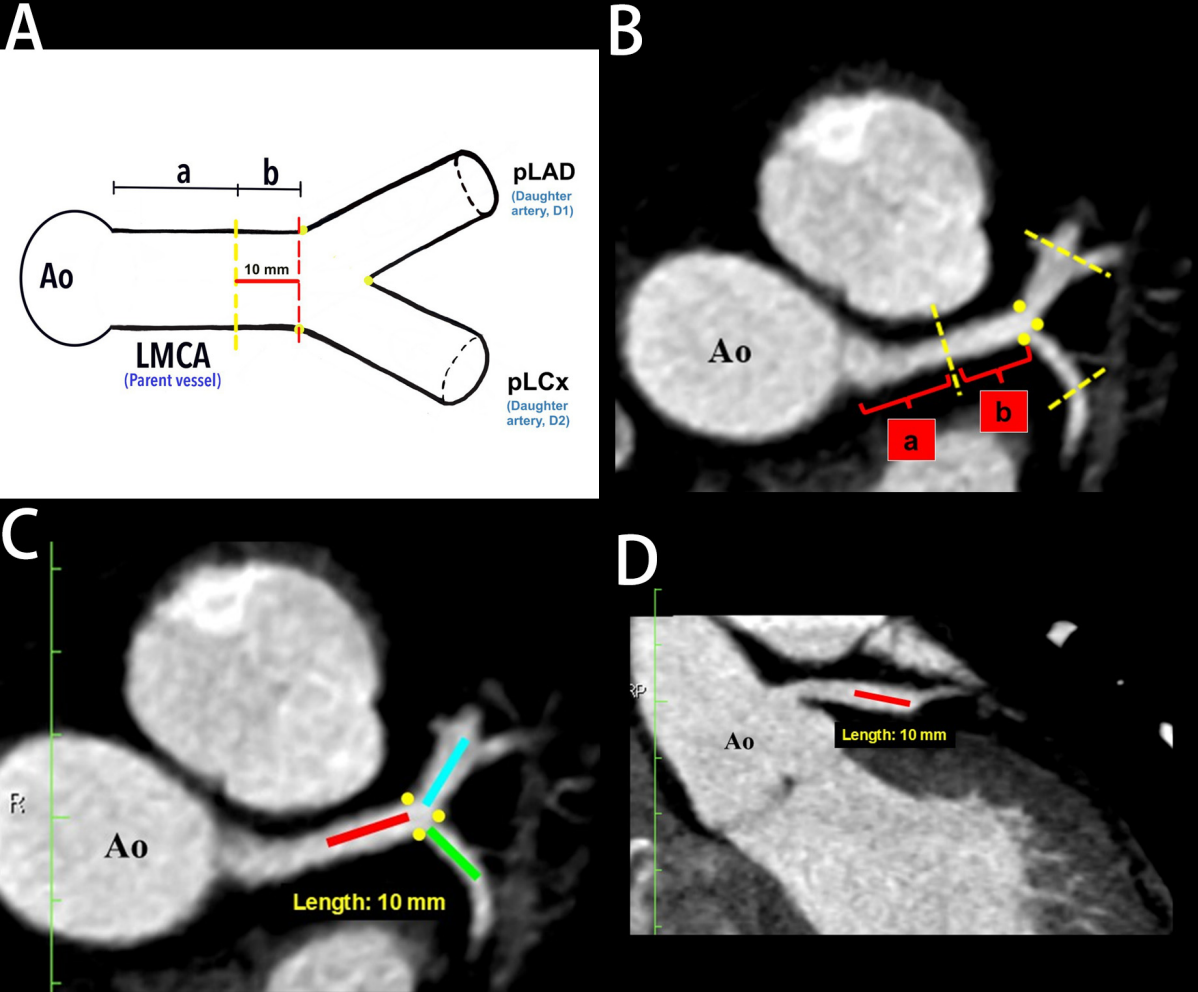
**The demarcation and identification of the left main coronary artery (LMCA) in a schematic diagram and in multiplanar reconstruction (MPR) images**. (A) Schematic diagram of proximal and distal portions of LMCA. (B) Proximal and distal portions of LMCA in MPR (axial view). (C) Left bifurcation region in MPR (axial view). (D) The LMCA in the MPR (coronal view). Yellow dots indicate the marking for the ostia of the daughter arteries, “*a*” indicates the proximal portion of LMCA, “*b*” indicates the distal portion of LMCA (red line), the blue line indicates 10 mm from pLAD ostium, and the green line indicates 10 mm from pLCx ostium. Ao, aorta; pLAD, proximal left anterior descending artery; pLCx, proximal left circumflex artery.

**Fig. 3.**
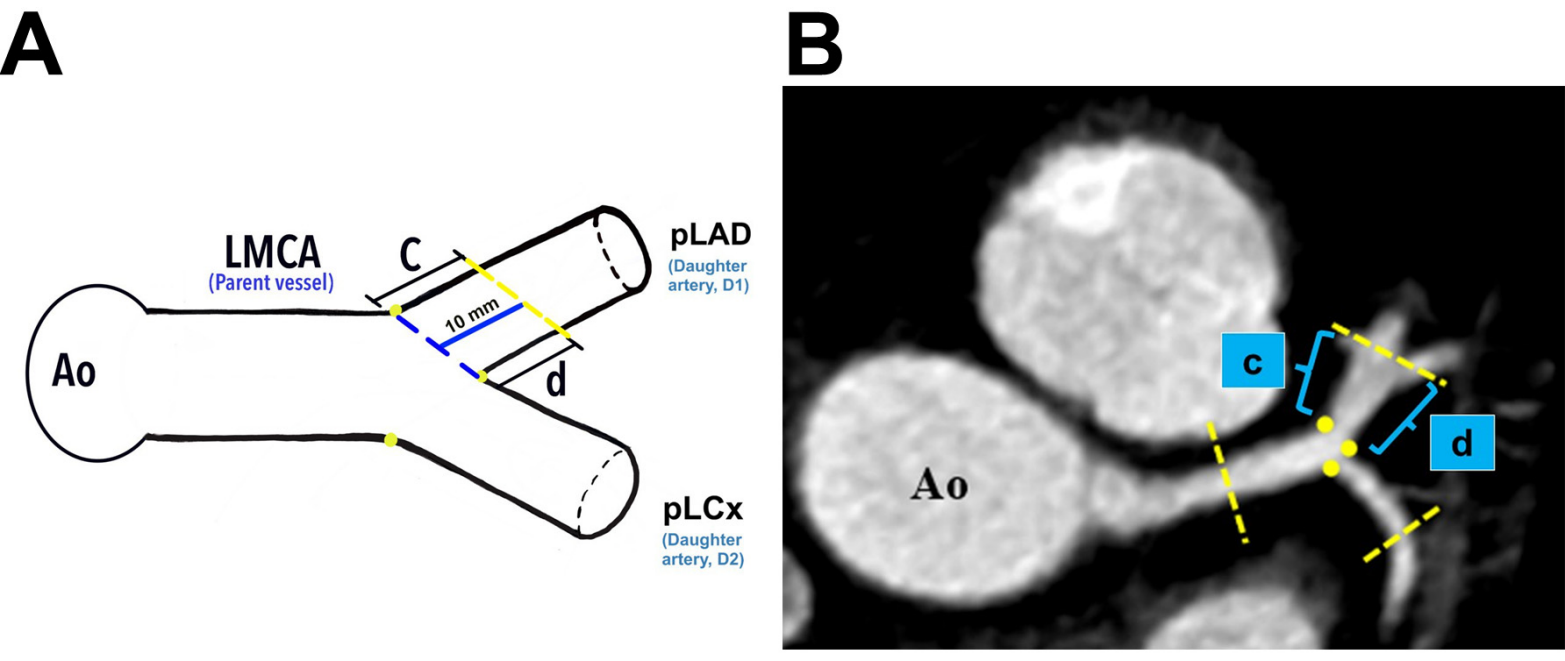
**The demarcation and identification of the proximal left anterior descending artery**. (A) Schematic diagram of the pLAD at a distance of 10 mm from its ostium (*blue line*). (B) The pLAD in MPR image (axial view). Yellow dots indicate the marking for the ostia of daughter vessels; “*c*” indicates the lateral wall, and “*d*” indicates the medial wall for pLAD. Ao, aorta; LMCA, left main coronary artery; pLAD, proximal left anterior descending artery; pLCx, proximal left circumflex artery; MPR, multiplanar reconstruction.

**Fig. 4.**
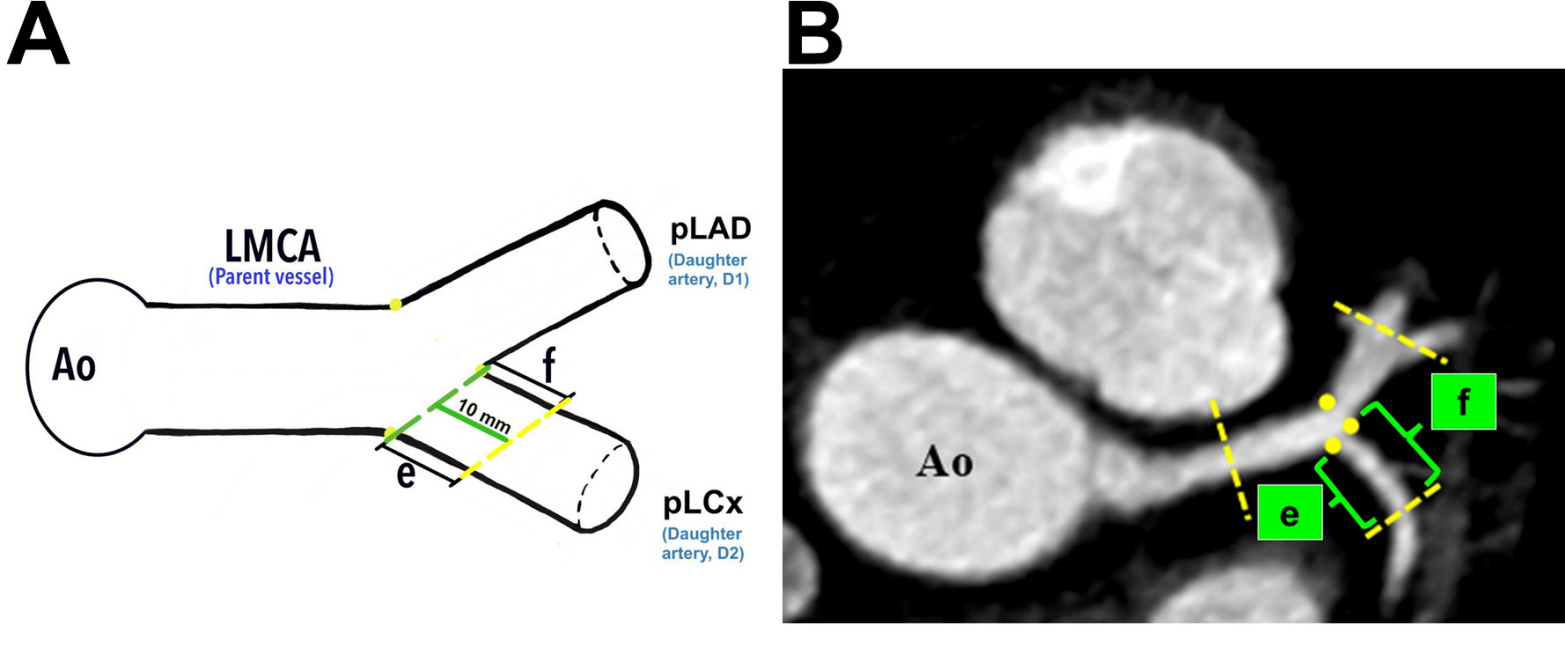
**The demarcation and identification of the proximal left circumflex artery**. (A) Schematic diagram of the pLCx at a distance of 10 mm from its ostium (*green line*). (B) The pLCx in an MPR image (axial view). Yellow dots indicate the marking for the ostia of daughter arteries; “*e*” indicates the lateral wall, and “*f*” indicates the medial wall for pLCx. Yellow dots represent the marking for the ostia of daughter vessels. Ao, aorta; LMCA, left main coronary artery; pLAD, proximal left anterior descending artery; pLCx, proximal left circumflex artery; MPR, multiplanar reconstruction.

Analysis of the LBR in this study was made according to the techniques described by Beton *et al*. (2017) [21]. Briefly, identification of the plaque location in the LMCA, proximal left anterior descending (pLAD), and proximal left circumflex (pLCx) were made at a specific distance as illustrated in the schematic diagrams (Fig. 2A, Fig. 3A, and Fig. 4A). The LMCA was taken as the parent vessel, whereas the pLAD and pLCx arteries were considered as the daughter vessels. The analysis was made within a 10 mm distance from the distal end of the LMCA and within a 10 mm distance from the ostia of the daughter vessels [21]. Images were reconstructed in a multiplanar reconstruction (MPR) format and analysed using the Centricity Picture Archiving and Communication System, Universal Viewer Version 5.0 (GE Healthcare, Chicago, IL, USA) (Fig. 2B–D, Fig. 3B, and Fig. 4B). A consistent setting was used to analyse the presence of the RI and LBR [20]. Those patients with the presence of RI were categorised into the “Experimental” group, and their data were analysed against patients with the absence of RI were categorised into the “Control” group (Fig. 1).

The LBR analysis investigated the association between the RI and atheroma formation near the LBR, with particular attention paid towards any predilection for the medial or lateral walls of the daughter arteries. In this study, the LMCA was divided into proximal (labelled “*a*”) and distal (labelled “*b*”) portions (Fig. 2A,B). The distal portion is defined as the area within a 10 mm distance from its distal-most ostium (depicted as the area between the red to yellow dotted lines, Fig. 2A). In the MPR images, the actual measurement of the distal portion of the LMCA taken at axial and coronal views are demonstrated in Fig. 2B–D.

Plaque analyses in the pLCx and pLAD arteries were made within a 10 mm distance from their ostia (i.e., the pLAD ostium was marked with a blue dotted line and the pLCx ostium was marked with a green dotted line, Fig. 3A and Fig. 4A). The actual measurement that was taken using MPR images is shown in Fig. 2C. To investigate the preference of plaque formation in either walls of the daughter vessels, the pLCx and pLAD were further subdivided into medial (labelled “*d*” for pLAD and “*f*” for pLCx) and lateral (labelled “*c*” for pLAD and “*e*” for pLCx) walls (Fig. 3A,B, Fig. 4A,B). A plaque was identified as a hyperdense region within the arterial wall that can either show a calcified, non-calcified, or mixed appearance. The presence of atheroma in the LMCA, pLAD and pLCx were documented.

### 2.2 Statistical Analysis 

Data were analysed using the statistical software package SPSS version 26.0 (SPSS Inc.; Chicago, IL, USA). Baseline characteristics of the study groups on RI and plaque depositions at the LMCA, pLAD, and pLCx were analysed using descriptive statistics, with frequencies expressed as percentages (%). The presence of the RI served as the independent variable. Meanwhile, plaque depositions at the LMCA, pLAD, and pLCx were the dependent variables. All data were made to compare between RI absence (control group) and RI presence (experimental group). Comparisons between groups were performed using the chi-square test. For cases with expected cell counts of less than five, the Fisher’s Exact test was applied. Binary logistic regression was used to adjust for age and to assess the risk association between RI and plaque deposition. A *p*-value < 0.05 was considered statistically significant.

## 3. Results

### 3.1 Study Groups

**Fig. 5.**
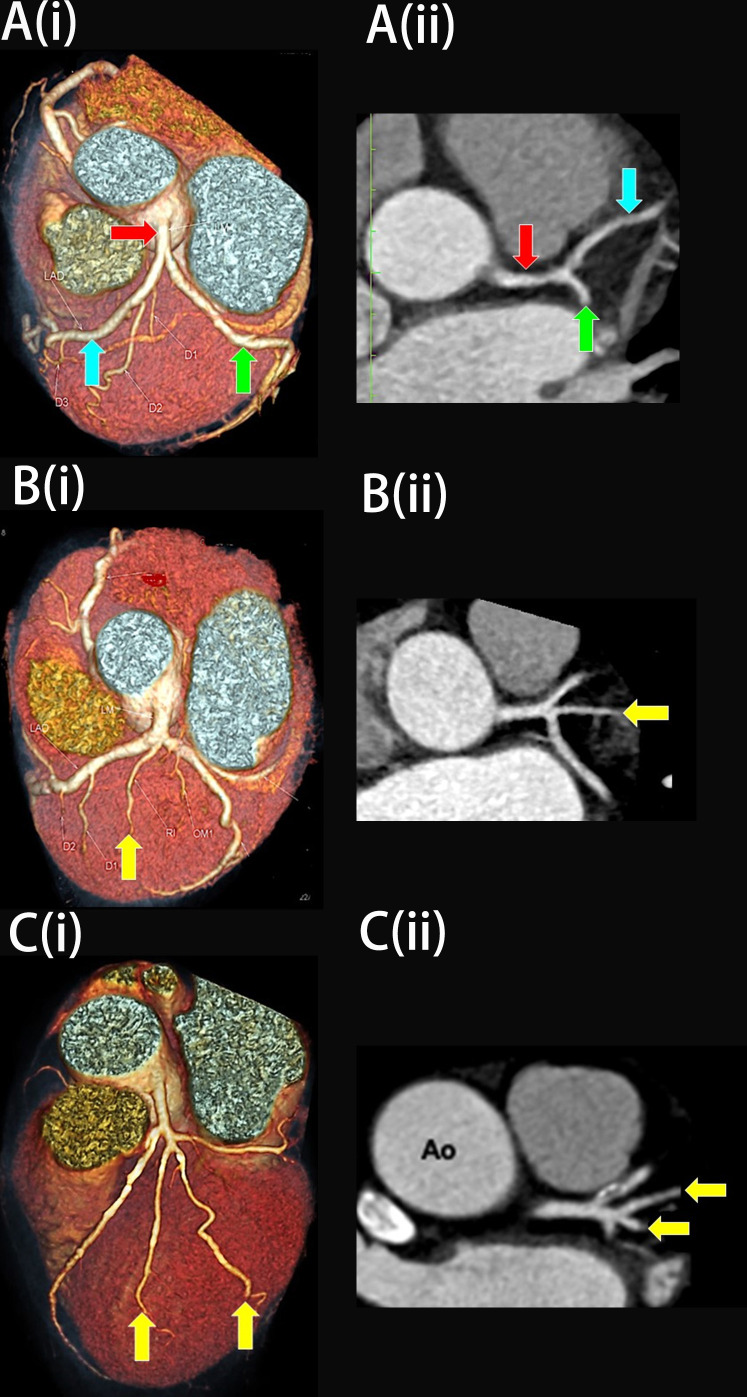
**The three variations of the left main bifurcation angle in cardiac computed tomography angiography images viewed in (i) Three-dimension volume rendering technique and (ii) Multiplanar reconstruction of the left bifurcation region (axial view)**. (A) Bifurcation. (B) Trifurcation with one single ramus intermedius (RI). (C) Quadfurcation with two vessels of RI. The red arrow indicates the left main coronary artery, the blue arrow indicates the left anterior descending artery, the green arrow indicates the left circumflex artery, and the yellow arrow indicates RI. Ao, Aorta.

**Fig. 6.**
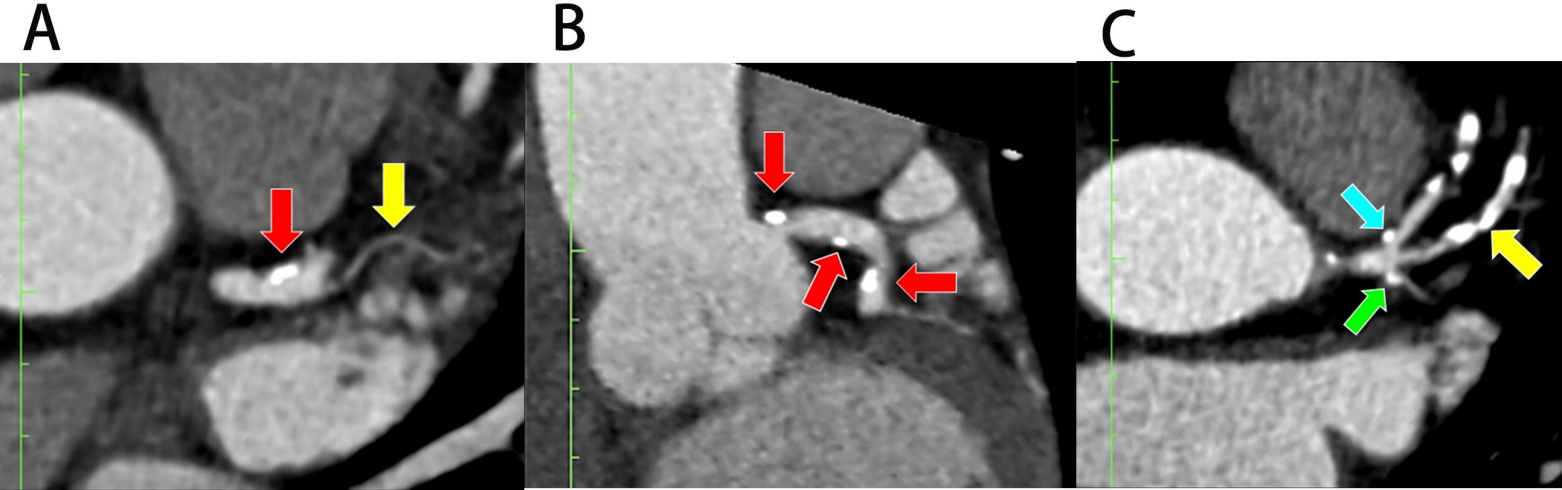
**Plaque deposition at the left bifurcation region in multiplanar reconstruction (MPR)**. (A) Plaque deposition at the distal portion of the left main coronary artery (LMCA) in the presence of ramus intermedius (RI) (axial view). (B) Plaque deposition at both proximal and distal portions of LMCA (coronal view). (C) Plaques at the lateral wall of proximal left anterior descending (pLAD) and proximal left circumflex (pLCx) arteries near their ostia in the presence of RI (axial view). The red arrow indicates LMCA, the blue arrow indicates pLAD, the green arrow indicates pLCx, and the yellow arrow indicates RI.

This study comprised of 139 females with low cardiovascular risk and no history of any cardiovascular diseases. Their ages were between 21 to 60. The findings revealed that 66.2% (n = 92) of the study participants had no aberrant RI artery at the LBR (Fig. 5A), while the remaining 33.8% (n = 47) had an RI (Fig. 1, and Fig. 6A,C). When present, the RI arteries were either in a single (30.2%, n = 42, Fig. 5B) or double vessel configurations (3.6%, n = 5, Fig. 5C). The inter-rater kappa for RI identification using MPR images was 1.0, demonstrating a very good agreement between the two investigators.

### 3.2 Plaque Deposition at the Left Bifurcation Region and its Association with RI

Table 1 highlights the distribution of plaques and their association with the RI. Analysis of the plaque at the LMCA, pLAD and pLCx was done using MPR images by two investigators with inter-rater kappa of 0.9 for LMCA, 0.7 for pLAD, and 0.8 for pLCx, respectively. There were 26 patients with plaques located at the LMCA; most of these were located at the distal portion (11.5%, n = 16), followed by both proximal and distal portions (6.5%, n = 9) and at the proximal (0.7%, n = 1) portion of the artery. Analysis showed that the RI had no significant relation to plaque development in these arterial portions individually, but concurrently influenced plaque development in both proximal and distal portions as shown in Fig. 6A,B. Multivariate analysis revealed that the RI posed an eight-fold increased risk for plaque deposition at both portions of the LMCA compared to patients without an RI (14.9%, n = 7, adjusted ratio (aOR) = 8.5, 95% confidence interval (CI) = 1.7, 43.0, *p* = 0.01, Table 1, LMCA).

**Table 1.** **Bivariate and multivariate analyses of patients with plaque deposition at the left bifurcation region involving the left main coronary, proximal left anterior descending, proximal left circumflex, and ramus intermedius arteries**.

Location of plaque deposition		Total (n = 139)	Ramus intermedius		Bivariate	Multivariate		
			(Control group)	(Experimental group)	*p-*value	aOR	95% CI	*p-*value
			(n = 92)	(n = 47)				
LMCA (n = 26)								
	Proximal (*a*) only	1 (0.7%)	1 (1.1%)	-	-	-	-	-
	Distal (*b*) only	16 (11.5%)	9 (9.8%)	7 (14.9%)	0.150	2.0	1.3, 5.5	0.244
	Both *(a and b*)	9 (6.5%)	2 (2.4%)	7 (14.9%)	0.006*	8.5	1.7, 43.0	0.010*
pLAD (n = 44)								
	Medial (*d*) only	2 (1.4%)	2 (2.2%)	-	-	-	-	-
	Lateral (*c*) only	24 (17.3%)	11 (12.0%)	13 (27.7%)	0.012*	3.5	1.1, 11.3	0.036*
	Both (*c and d*)	18 (12.9%)	7 (7.6%)	11 (23.4%)	0.025*	4.3	2.1, 17.4	0.038*
pLCx (n = 27)								
	Medial (*f*) only	2 (1.4%)	1 (1.1%)	1 (2.1%)	-	-	-	-
	Lateral (*e*) only	12 (8.6%)	5 (5.4%)	7 (14.9%)	0.224	0.8	0.2, 4.4	0.872
	Both (*f and e*)	13 (9.4%)	6 (6.5%)	7 (14.9%)	0.478	3.7	1.2, 11.4	0.769
RI		14 (30.0%)	-	14 (30.0%)	-	-	-	-

Values are n (%). *Indicates *p* < 0.05, aOR, adjusted odds ratio using binary logistic regression test with the absence of RI (Control group) as the reference. “*a*” indicates the proximal portion of LMCA, “*b*” indicates the distal portion of LMCA, “*c*” indicates the lateral wall of pLAD, “*d*” indicates the medial wall of pLAD, “*e*” indicates the lateral wall of pLCx, and “*f*” indicates the medial wall of pLCx. CI, confidence interval; LMCA, left main coronary artery; pLAD, proximal left anterior descending artery; pLCx, proximal left circumflex artery; RI, ramus intermedius.

There were 44 patients who had plaques within the 10 mm distance from the ostium of pLAD. Most of the plaques were formed along the lateral wall of this artery (17.3%, n = 24), followed by plaques along both lateral and medial walls (12.9%, n = 18) and plaques along the medial wall only (1.4%, n = 2). The presence of the RI tripled the risk for plaque deposition along the lateral wall in pLAD (27.7%, n = 13, aOR = 3.5, 95% CI = 1.1, 11.3, *p* = 0.036) and added a four-fold increased risk for plaque formation involving both lateral and medial walls of this artery (23.4%, n = 11, aOR = 4.3, 95% CI = 2.1, 17.4, *p* = 0.038; Table 1, pLAD). Fig. 6C demonstrated a plaque at the lateral wall of pLAD.

There were 27 patients with plaque deposition within the 10 mm distance from the ostium of the pLCx (Fig. 6C), but the bivariate analysis did not show that the RI had any association with plaque deposition at any of the walls of this artery (Table 1, pLCx). In addition, 14.9% (n = 7) of patients with an RI were noted to have plaque deposition in the pLCx (Table 1, pLCx and Fig. 6C). Out of 47 patients that had an RI, 14 (30.0%) demonstrated stenotic plaques in their RI (Table 1, RI and Fig. 6C). Fig. 6C demonstrated a plaque involving the RI.

## 4. Discussion

Atherosclerosis is a multifactorial condition influenced by biochemical and biomechanical factors [8, 22, 23]. Biomechanical elements, such as bifurcation, additional branch points, and increased epicardial curvature alter arterial geometry and disrupt haemodynamic blood flow [5, 6, 11, 22]. This study sought to investigate the role of the RI in atherosclerotic plaque deposition within the LBR. The results suggested that anatomical variations such as RI may significantly influence the development of atherosclerosis even in low-risk individuals. These findings emphasise the importance of incorporating coronary artery anatomy into cardiovascular risk assessment.

### 4.1 Prevalence and Variations of RI 

The prevalence of RI in this cohort was 33.8%, which aligns with previous studies that report incidence rates between 20% to 35% [21, 24, 25, 26]. Populations from China and Thailand have noted a higher incidence, ranging from 65% to 69% [14, 27, 28]. The RI was identified through various methods, including CCTA, intravascular ultrasound, coronary angiography, and examination of cadaveric hearts [14, 21, 24, 25, 26, 27, 28]. It can present as a single, double, or triple vessel formation, leading to the development of a trifurcation, quadfurcation and pentafurcation at the LBR [29, 30, 31, 32, 33, 34, 35, 36]. Research by Patel JP *et al*. (2016) [32] and Ogeng’o JA *et al*. (2014) [33] on formalin-fixed hearts found that a bifurcation was the most frequent termination pattern for the LBR, followed by trifurcation, quadfurcation, and pentafurcation. The current study demonstrated that a single RI was more common than a double RI, consistent with the findings from other research [31, 34, 35, 36]. This study did not observe triple RI, likely due to its rarity, as it has been reported in only 1% to 3% of cases [31, 32, 33].

### 4.2 The Association between RI and Plaque Deposition at the LBR

The overall patterns of plaque deposition in patients with RI (experimental group) and without RI (control group) were generally similar. However, the presence of the RI was associated with a two-fold increase in the risk of plaque deposition at the LMCA and pLAD, particularly along the lateral wall. Although plaques were observed in some patients at the pLCx, no significant association with the RI was identified. Additionally, this study identified that the RI was a site prone to plaque deposition. It is hypothesised that the presence of the RI introduces an additional branch point resulting, in tri-, quad-, or pentafurcation that further widens the LAD-LCx curvature [11]. This altered geometry of LBR influences ESS, leading to endothelial damage and atheroma formation [37].

**Fig. 7.**
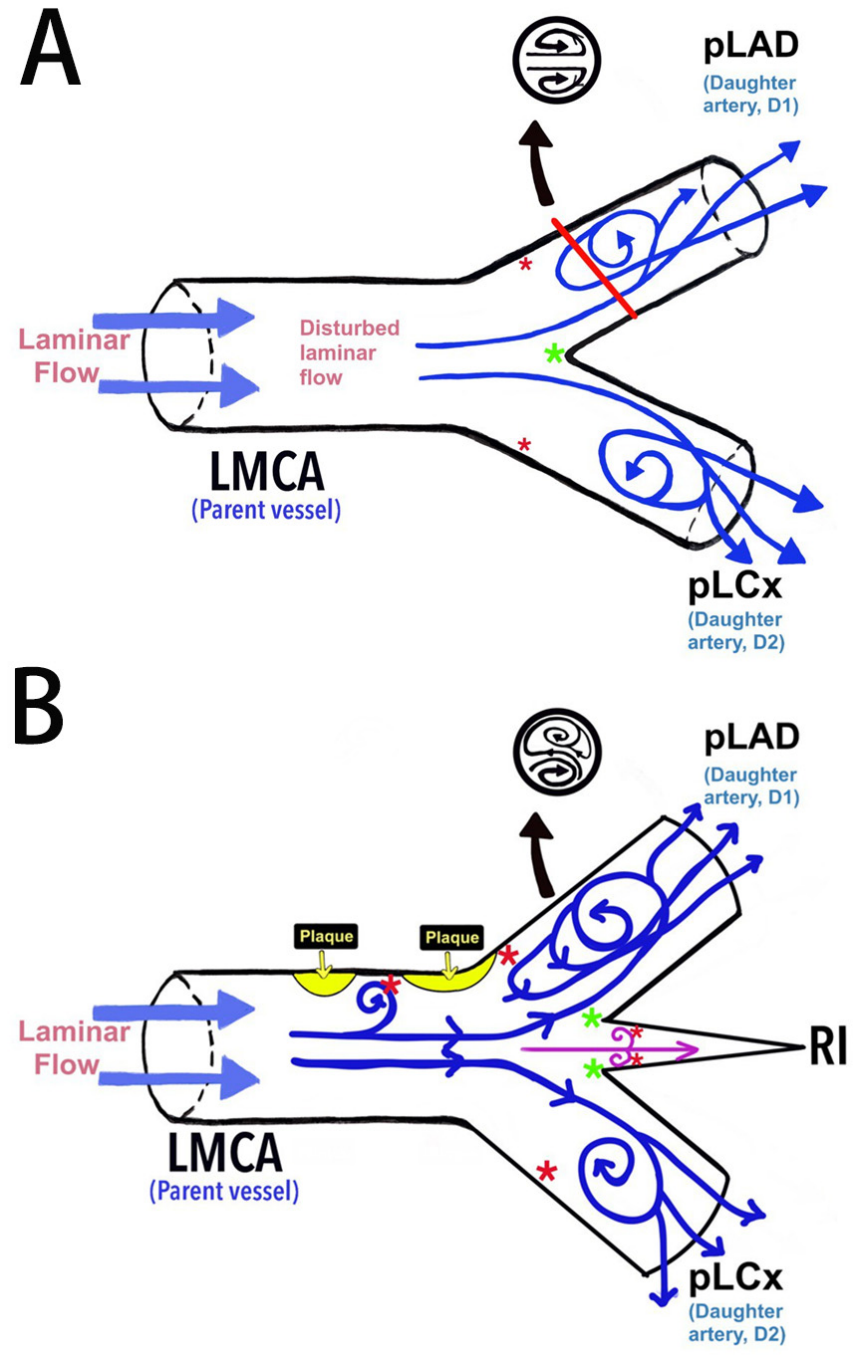
**The haemodynamic blood flow in the left bifurcation region**. (A) Schematic diagram of the haemodynamic blood flow and its association with endothelial shear stress (ESS). (B) The proposed model of the left bifurcation region in the presence of RI as an additional branch point and its association with ESS according to the findings of this current study. *Red asterisk indicates low ESS and *green asterisk indicates high ESS. LMCA, left main bifurcation artery; pLAD, proximal left anterior descending artery; pLCx, proximal left circumflex artery; RI, ramus intermedius.

In the absence of the RI, the baseline configuration of the LBR shows a disrupted laminar flow upon reaching the bifurcation point. The laminar flow turns to a swirl along the lateral wall of LAD and LCx near their ostia (Fig. 7A) [8, 37]. Previous studies have shown that a T-shaped LMBA with a LAD-LCx angle of >90° carries a higher risk for plaque formation compared to a Y-shaped LMBA where the LAD-LCx angle was <90° [11, 38]. When RI is present, the angle at the LBR would widen even further [11, 37]. Rabbi MF *et al*. (2020) [37] modelled patient-specific geometries with bifurcation angles of 70°, 95°, and 135°, and trifurcation angles of 90°. The computational fluid dynamics analyses showed that a lower ESS occurred at the widest angle, especially near its ostia. The trifurcation geometries exhibited an even a lower ESS than the bifurcation [37].

A similar study by Singhal *et al*. (2024) [39] utilised three dimensional patient-specific coronary models constructed from CCTA images to measure ESS in trifurcation geometries. The study found that the presence of the RI created a larger area of low ESS along the lateral wall of the pLAD. Schultz *et al*. (2023) [10] demonstrated that CCTA-based analyses of ESS in normal coronary arteries showed that low ESS is more prevalent near the pLAD ostium (lateral wall), with high ESS concentrated at the carina (medial wall), as shown in Fig. 7A. The pLAD exhibited the widest range of ESS variation (i.e., oscillatory and high ESS) compared to the LMCA and pLCx [10, 40, 41]. These findings were consistent with studies by El Zayat *et al*. (2021) [25] and Zhang *et al*. (2023) [14], demonstrating that the pLAD was more susceptible to plaque deposition in the presence of the RI. Both studies suggested it was due to low longitudinal strain in the LCx [13, 25]. However, this phenomenon requires further investigation.

In addition to the pLAD, the RI also increased the risk of developing plaque at the LMCA and in the RI itself. These findings were consistent with Pourafkari *et al*. (2023) [42], who found that the plaque caused varying degrees of stenosis in these vessels. The involvement of the LMCA is particularly concerning, as it serves as the primary arterial supply to the myocardium of the left side of the heart [42, 43]. However, isolated plaque deposition in the LMCA is rare, and it typically occurs alongside the LAD and/or the LCx involvement, resulting in two or three-vessel disease [44, 45]. Findings from the current study postulated that plaque formation in the LMCA could be driven by plaque progression in the daughter vessels, particularly the pLAD. Low ESS at the proximal shoulder of the plaque likely induces endothelial damage in the LMCA, initiating plaque formation [7, 22, 41, 46].

Previous research demonstrated the presence of RI associated with plaque deposition at the pLAD and LMCA [13, 25, 42]. However, its relations with specific types of plaque (i.e., calcified, non-calcified, and mixed) require further investigation. The presence of the RI exacerbates haemodynamic disturbances, possibly forming a Lagrangian coherent structure with three elliptical ridges that affect the lateral wall of the daughter vessels, particularly at the pLAD (Fig. 7B) [23, 41]. Therefore, the current study suggests that the RI may represent a non-modifiable risk factor for coronary artery disease. The hypothesis that RI induces extreme ESS (i.e., oscillatory and high ESS) requires further investigation beyond the scope of the study.

### 4.3 Study Limitations

This study has several limitations. First, the study is limited by its single-centre and retrospective nature, which may limit the generalisability of the findings to different populations. Second, the sample size was relatively small, which reduced its statistical power. Future studies should include a larger sample size to improve test efficiency. Third, the study did not quantify ESS levels in the LBR, which would have provided additional support for the study’s hypotheses. Fourth, the characteristics of the plaques, including their type and vulnerability, were not identified, leaving a gap in understanding the specific atherosclerosis changes. Finally, the left main bifurcation angle between the LAD – LCx was not measured, which may provide insights into its influence on intracoronary haemodynamics and ESS.

## 5. Conclusions

Key findings from this study suggest that in the absence of all other cardiovascular risk factors, the presence of an RI may significantly contribute to plaque deposition at the LMCA, pLAD, and in the RI itself. These findings highlight the importance of coronary artery anatomy in cardiovascular risk assessment. Further research is needed to better understand the haemodynamic effects of the RI and its role in atherosclerosis.

## Data Availability

The datasets used and/or analysed during the current study are available from the corresponding author on a reasonable request.

## Abbreviations

aOR, adjusted odds ratio; Ao, aorta; CCTA, cardiac computed tomography angiography; CI, confidence interval; ESS, endothelial shear stress; LAD, left anterior descending; LBR, left bifurcation region; LCx, left circumflex; LMBA, left main bifurcation angle; LMCA, left main coronary artery; MPR, multiplanar reconstruction; pLAD, proximal left anterior descending artery; pLCx, proximal left circumflex artery; RI, ramus intermedius.
